# Augmented reality for interventional oncology: proof-of-concept study of a novel high-end guidance system platform

**DOI:** 10.1186/s41747-018-0054-5

**Published:** 2018-07-31

**Authors:** Marco Solbiati, Katia M. Passera, Alessandro Rotilio, Francesco Oliva, Ilaria Marre, S. Nahum Goldberg, Tiziana Ierace, Luigi Solbiati

**Affiliations:** 1 R&D Unit, R.A.W. Srl, Busto Arsizio, VA Italy; 2 Department of Radiology, Hadassah Hebrew University Medical Centre, Jerusalem, Israel; 3 Department of Radiology, Beth Israel Deaconess Medical Center, Boston, MA USA; 4 Department of Radiology, Humanitas Clinical and Research Center, Rozzano, Milan, Italy; 5 Department of Biomedical Sciences, Humanitas University, Pieve Emanuele, Milan, Italy

**Keywords:** Augmented reality, Liver, Neoplasms, Radiology, interventional, Tomography, x-ray computed

## Abstract

**Background:**

To assess the feasibility of a novel system that uses augmented reality to guide interventional oncology procedures.

**Methods:**

This study was conducted in accordance to the guidelines of the local institutional review boards. Evaluation of an augmented reality system based upon a tablet, a needle handle and a set of markers was performed in three experimental models. Initially, a male anthropomorphic trunk phantom equipped with five polyvinyl chloride bars (two of 16 cm in length and 3 cm in diameter and four of 45, 30 or 20 cm in length and 2 cm in diameter) was used to study the accuracy of the system without respiratory motion or tissue compression. Next, small metallic targets were placed in a porcine model to evaluate how respiration affects the system accuracy. Finally, the performance of the system on a more complete model, a cadaver with liver metastasis, was tested.

**Results:**

In all experimental settings, extremely high targeting accuracy of < 5 mm in all cases was achieved: 2.0 ± 1.5 mm (mean ± standard deviation) for the anthropomorphic model, 3.9 ± 0.4 mm for the porcine model, and 2.5 mm and 2.8 mm for the two metastases in the cadaver model.

**Conclusions:**

Augmented reality can assist with needle guidance with great target accuracy for interventional procedures by simultaneously visualising three-dimensional reconstructed anatomical structures, tumour targets and interventional devices on a patient’s body, enabling performance of procedures in a simple and confident way.

## Key points

- A new guidance system (Endosight) for interventional procedures based on augmented reality has been tested.
- To assess the accuracy of the system we performed a three-step experiment using different models: an anthropomorphic trunk model; a porcine model; and a cadaver.
- Based upon our experiments, the system is precise and reliable, with targeting accuracy always < 5 mm.
- This new system can guide interventional procedures without the need for intraprocedural imaging.

## Background

Interventional oncology, the youngest and most rapidly growing offshoot of interventional radiology, has successfully established itself as an essential and independent pillar within the firmament of multidisciplinary oncologic care, alongside medical, surgical and radiation oncology [1]. Interventional oncology deals with the diagnosis and treatment of cancer and cancer-related problems using targeted minimally invasive procedures performed under image guidance. Among these, during the past 25 years, image-guided thermal ablation has been validated and increasingly used for the treatment of neoplastic diseases because of its low invasiveness, efficacy, repeatability and low cost [2]. However, precise image guidance is critical to the success of interventional oncology procedures. Navigational tools can enhance the interventional precision by improving localisation of devices in relation to the target. Currently available navigational tools for interventional radiology include electromagnetic, optical, laser and robotic guidance systems as well as image fusion platforms [3]. Such automation, navigation and visualisation tools may eventually optimise needle-based ablation procedures and decrease variability among operators, thus facilitating the diffusion of novel image-guided therapies [4].

The specifications of the various navigation systems are based on indicating the position of a biopsy needle or an ablation applicator using point-to-point navigation [5]. In other words, when the operator points within the interventional field using a specified indicating probe, the corresponding location is indicated on the magnetic resonance (MR)/computed tomography (CT) scans. Accordingly, interventional oncologists are required to create a three-dimensional (3D) mental image of the MR/CT views to match the interventional field. In addition, they must frequently alternate their gaze between the interventional field and the instrumentation screen. To overcome this difficulty, several navigation systems have been developed using augmented reality techniques [5]. In fact, augmented reality can allow the operator to see 3D virtual objects superimposed upon the real world and not on a different screen.

We have developed a novel guidance system based on augmented reality. A tablet personal computer is used for visualisation. The patient’s body is captured by the back-face camera of the tablet. Three-dimensional images of body structures (liver, vessels, lesions, etc.) are extracted from preoperative CT (or MR) scans and are superimposed on the video image of the patient. When viewed from various directions around the patient, body structures are displayed with corresponding angles as viewed from the camera direction, thus giving the interventional oncologist the sensation of seeing through the patient. In addition, the needle is also virtualised in its real-time position with the distance between the needle tip and the geometric centre of the target displayed to provide precise real-time guidance for lesion targeting.

The aim of this paper is to describe the Endosight system (Endosight, R.A.W. S.r.l., Milan, Italy) and to present preliminary results of its application in phantom and porcine models as well as in a human cadaver.

## Methods

This study was conducted in accordance to the guidelines of the local institutional review boards.

A three-step protocol study was performed, as follows:

(i) to investigate the targeting accuracy in a rigid setting without organ motion, experiments were performed using a custom-made phantom of the trunk;
(ii) to investigate the navigation system in vivo (considering respiration effect), a porcine trial was conducted under two different respiratory conditions, with and without breathing control;
(iii) finally, to investigate the system accuracy in a quasi-clinical scenario, a cadaver with liver metastases was used.

We used a 5 mm accuracy threshold, defined as the distance between the geometric centre of the target and the needle tip, based upon key clinical considerations. In fact, most thermal ablations are performed on tumours with a diameter in the range of 1–3 cm [6, 7]. Thus, a guidance system must provide a targeting accuracy in the range of 5 mm from the geometric centre of the target in order to allow to achieve complete tumour necrosis in a single treatment session and to avoid destroying too much healthy tissue.

In all the three experimental settings, we computed accuracy and we verified that this limit was fulfilled.

### Endosight system

**Fig. 1:**
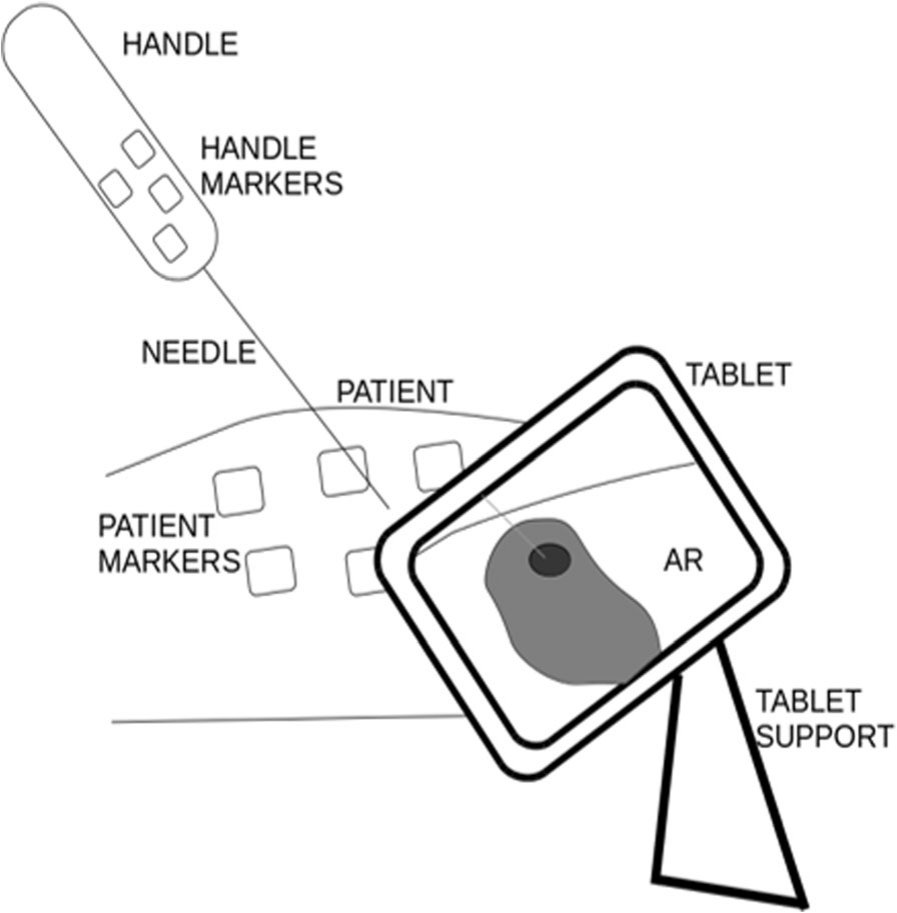
Endosight system components. *AR* augmented reality

To obtain augmented reality, a customised needle handle manufactured by using geometrically configured, fused deposition modelling technology attached to a 17 cm needle and markers glued on top of the handle were used. In addition, radiopaque tags were applied on patient’s skin to serve as fiducial markers. The augmented reality was then displayed by a tablet (Microsoft Surface Pro 4 with a 12-megapixel camera, Microsoft, Redmond, WA, USA) attached to a stable tripod platform (Fig. 1).

**Fig. 2:**
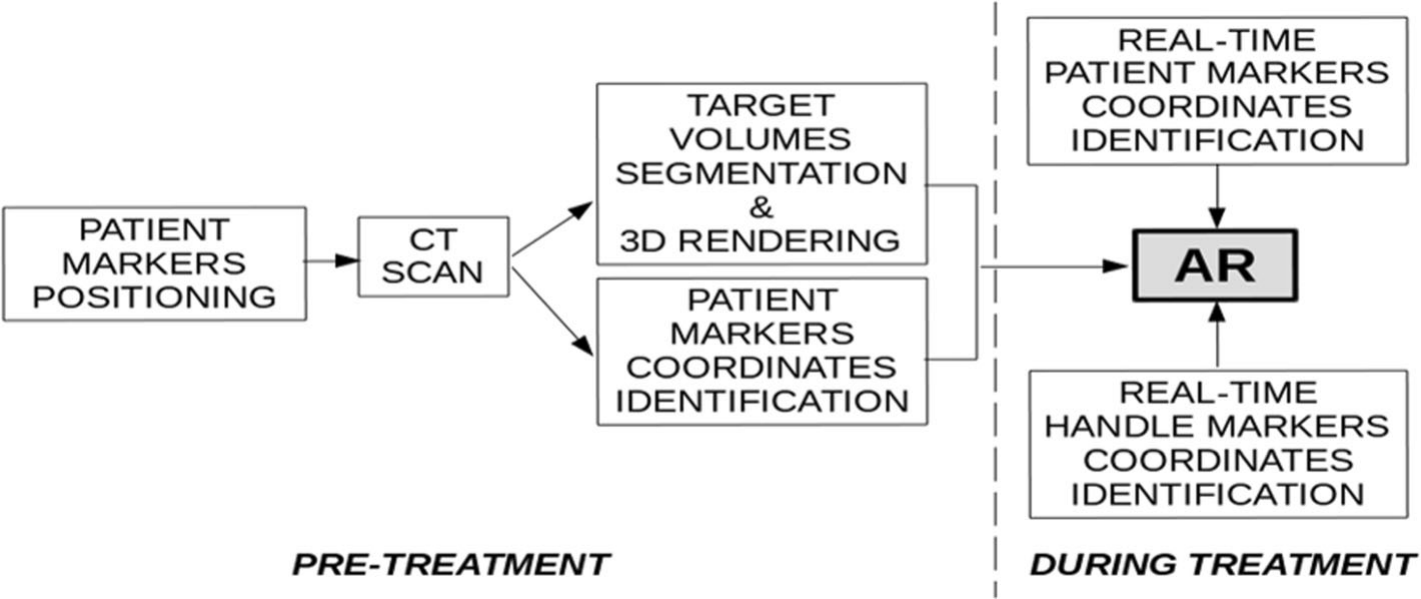
Interventional oncology workflow using Endosight. *CT* computed tomography, *AR* augmented reality

The workflow to generate augmented reality during the procedure is summarised in Fig. 2. Before intervention, radiopaque skin markers should be positioned on the patient’s body. Next, a CT scan is acquired. The CT images are then processed: automatic image segmentation algorithms are used to automatically obtain outline of target volumes. The fiduciary markers on the patient skin are segmented as well, with their position coordinates extracted using a principal component algorithm. During the intervention, the pre-treatment information, together with the real-time position of needle handle and patient markers, permits computation (the software used to create the augmented reality is: Unity 2017, f 1.1) and display of the augmented reality superimposed upon the visualised background of the interventional procedure. In fact, the proprietary software recognises a geometric configuration of fiducial markers on both the ablation guide handle and the patient. When the distance between the needle tip and the geometric centre of the target is 0, the needle is properly positioned at the centre of the intended target.

### Anthropomorphic trunk model protocol

**Fig. 3:**
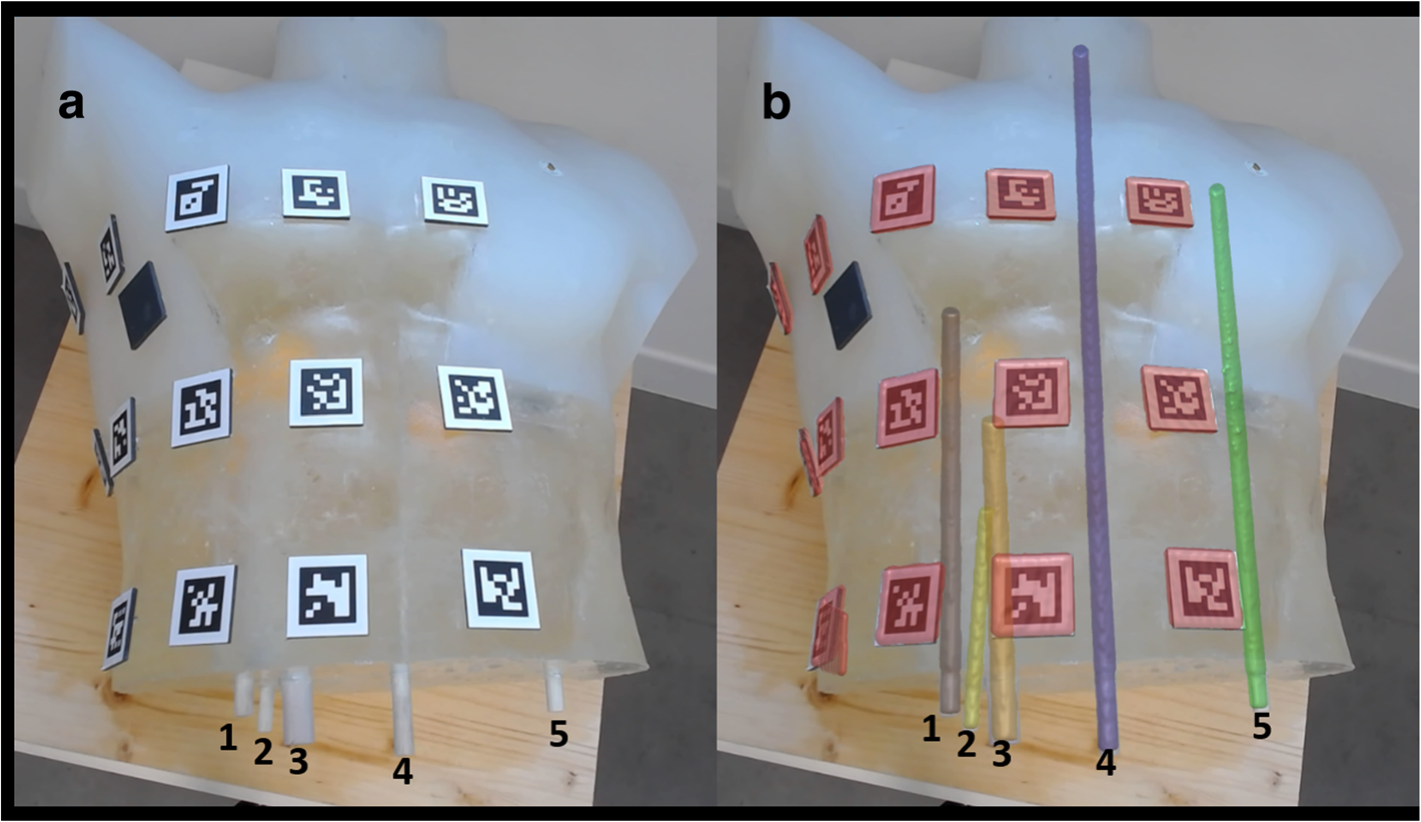
Anthropomorphic chest model without augmented reality (**a**) and with augmented reality (**b**). The augmented reality shows the segmented objects superimposed to the reality: markers are shown in *red*, while the five bars are shown in *grey*, *yellow*, *orange*, *purple* and *green*

A semi-transparent silicone anthropomorphic phantom (50 × 34 × 27 cm) was constructed pouring a silicone material into a trunk gypsum mould (Fumagalli & Dossi, Milan, Italy). Multiple targets were positioned inside the phantom. These consisted of five polyvinyl chloride bars (two of 16 cm in length and 3 cm in diameter and four of 45, 30 or 20 cm in length and 2 cm in diameter). When the silicone hardened, the phantom was extracted by the mould. Thirty 3.5 × 3.5 cm radiopaque squares were placed on the phantom surface to serve as fiduciary markers, as shown in Fig. 3.

The phantom underwent CT using a 64-slice unit (LightSpeed, General Electric Healthcare Milwakee, WI, USA), with the following technical parameters: collimation = 3–5 mm; reconstruction interval = 2 mm; matrix = 512 × 512; in-plane pixel size = 0.48–0.86 mm; 120 kVp; 200 mAs; gantry rotation time = 0.8 s; and pitch = 1.75. Targets and markers were segmented and reconstructed in 3D with marker coordinates automatically derived.

To compute accuracy, we manually measured the distance between any pair of targets (geometric centre of bar) both on the actual phantom and on the augmented reality images obtained at three different tablet camera-phantom distances (30 mm, 40 mm, and 50 mm).

### Porcine model protocol

Institutional animal care and use committee approval was obtained for the use of a swine model of this study. A female Yorkshire swine (aged 6 months old, weighing 93 kg; Mizra, Lahav, Israel) was studied with the supervision of the division of animal facility authority at Hadassah Hebrew University (Jerusalem, Israel). The animal received appropriate care from properly trained professional staff in compliance with both the Principles of Laboratory Animal Care and the Guide for the Care and Use of Laboratory Animals, approved by the Animal Care and Use Committees of The Hebrew University and in accordance with National Institutes of Health guidelines. The swine was initially anaesthetised with ketamine injection United States Pharmacopeia (Ketaset CIII, Fort Dodge Animal Health, IA, USA) 15 mg/kg, xylazine 2 mg/kg (Kepro, Deventer, The Netherlands), propofol 2% (one injection of 5 mL). Thereafter, the animal underwent endotracheal intubation followed by inhaled anaesthetic isofluorane (5% induction, 1.5–2.5% maintenance). No paralytics were used during the procedure.

**Fig. 4:**
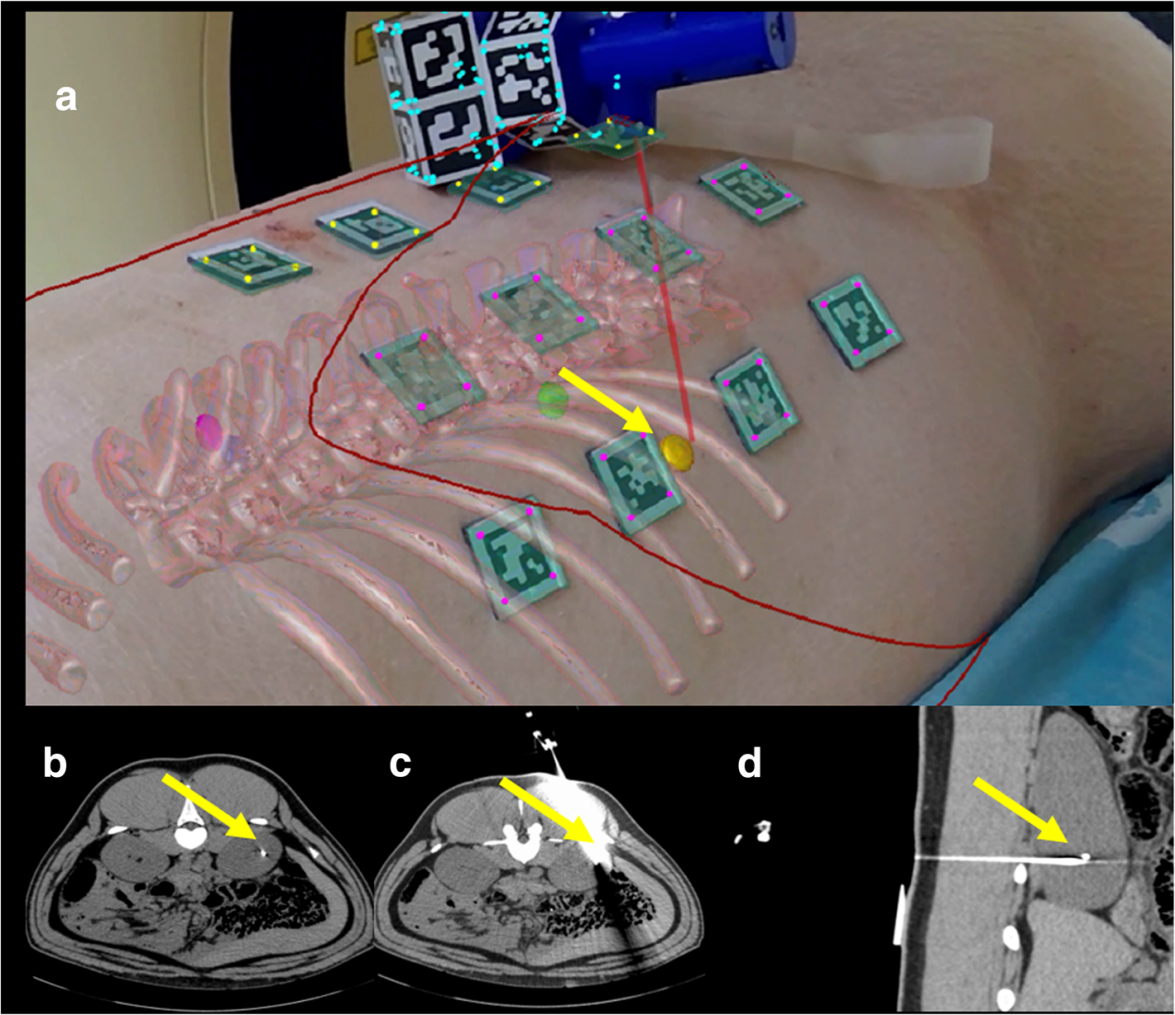
Porcine model with augmented reality during needle insertion in the right kidney target (**a**). The augmented reality shows the target in *yellow* and the needle in *red*. The *curved red lines* in the image represent the outlines on the porcine model. Correspondent axial CT images before (**b**) needle insertion and axial (**c**) and sagittal (**d**) CT images after needle insertion. The *yellow arrows* show the target and the needle positions in all the images and confirm that in all the cases the target was reached. All images refer to the test performed with breathing control

**Fig. 5:**
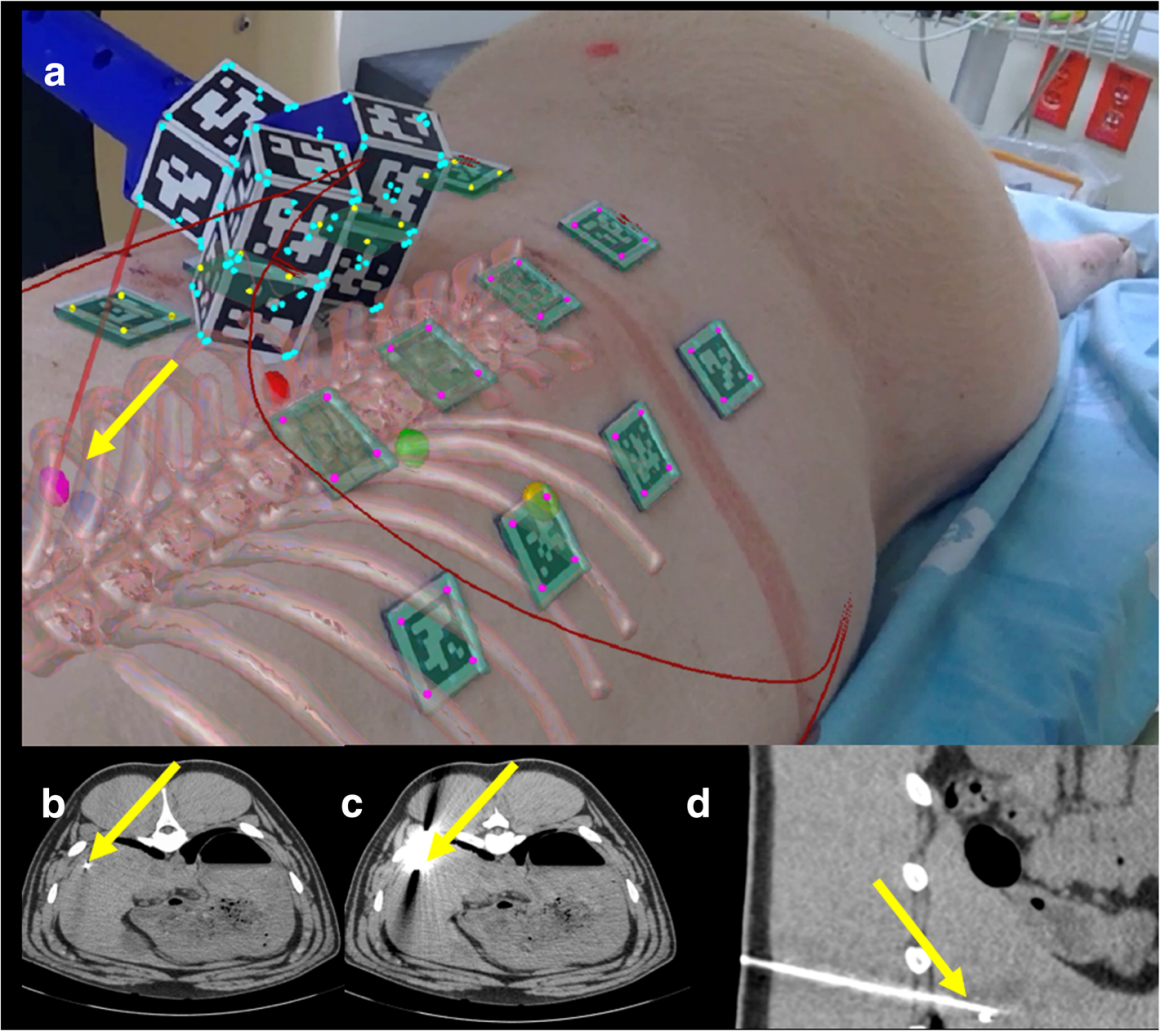
Porcine model with augmented reality during needle insertion in one liver target (**a**). The augmented reality shows the target in *magenta* and the needle in *red*. The *curved red lines* in the image represent the outlines on the porcine model. Correspondent axial CT images before (**b**) needle insertion and axial (**c**) and sagittal (**d**) CT images after needle insertion. The *yellow arrows* show the target and the needle positions in all the images and confirm that in all the cases the target was reached. All images refer to the test performed with breathing control

The swine was placed on the CT table in prone position for kidney targeting and in the decubitus position for liver targeting. Using a coaxial 18-G needle technique (BrachyStar needles, Bard, Covington, GA, USA) under direct CT guidance, small (2 × 1 mm) metallic markers were embedded in three different anatomic locations (one in the kidney, two in the liver), as previously reported [8]. Twelve radiopaque markers were placed on the pig’s skin (Figs. 4a and 5a).

Once all targets were placed, a CT scan was performed with respiration suspended at maximum expiration. Then, the volume targets (skin, bone, targets, markers) were reconstructed by using reconstruction proprietary algorithms. Next, the needle was inserted using Endosight augmented reality guidance into the pig reaching each target centre indicated as a distance equal to 0 by the augmented reality system. Once completed, an expiratory breath-hold CT scan with the inserted needle was performed to verify the correspondence between what was shown by the augmented reality system and the result of the CT scan.

All CT scans were performed using a dual-layer 64-detector CT prototype, model iCT SDCT (Philips Healthcare, Cleveland, OH, USA). The scanning parameters were: 120 kVp; 200 mAs; gantry rotation time = 0.33 s; collimation = 40 mm (64 × 0.625 mm); and pitch = 0.984. Scans were reconstructed as contiguous slices of 1-mm thickness, using a standard soft-tissue convolution kernel.

The accuracy was simply measured as the distance between the geometric centre of the target and the needle tip on CT images. Two different conditions were tested:

(i) with breathing control – the needle was inserted in the pig when it was in the maximum expiratory phase that corresponded to the same respiratory phase as that registered during the initial CT scan;
(ii) without breathing control – with the pig breathing freely and the respiratory phase was not known.

### Cadaver model protocol

This study was performed at the Simulation Center of Humanitas University (Pieve Emanuele – Milan, Italy) on a 73-year-old female torso cadaver with a history of liver metastases donated to science and obtained from medcure.org (Medcure, Orlando, FL, USA). To investigate location and number of liver metastases, the cadaver underwent ultrasound (US) scan (My Lab Gamma, Esaote, Genoa, Italy).

**Fig. 6:**
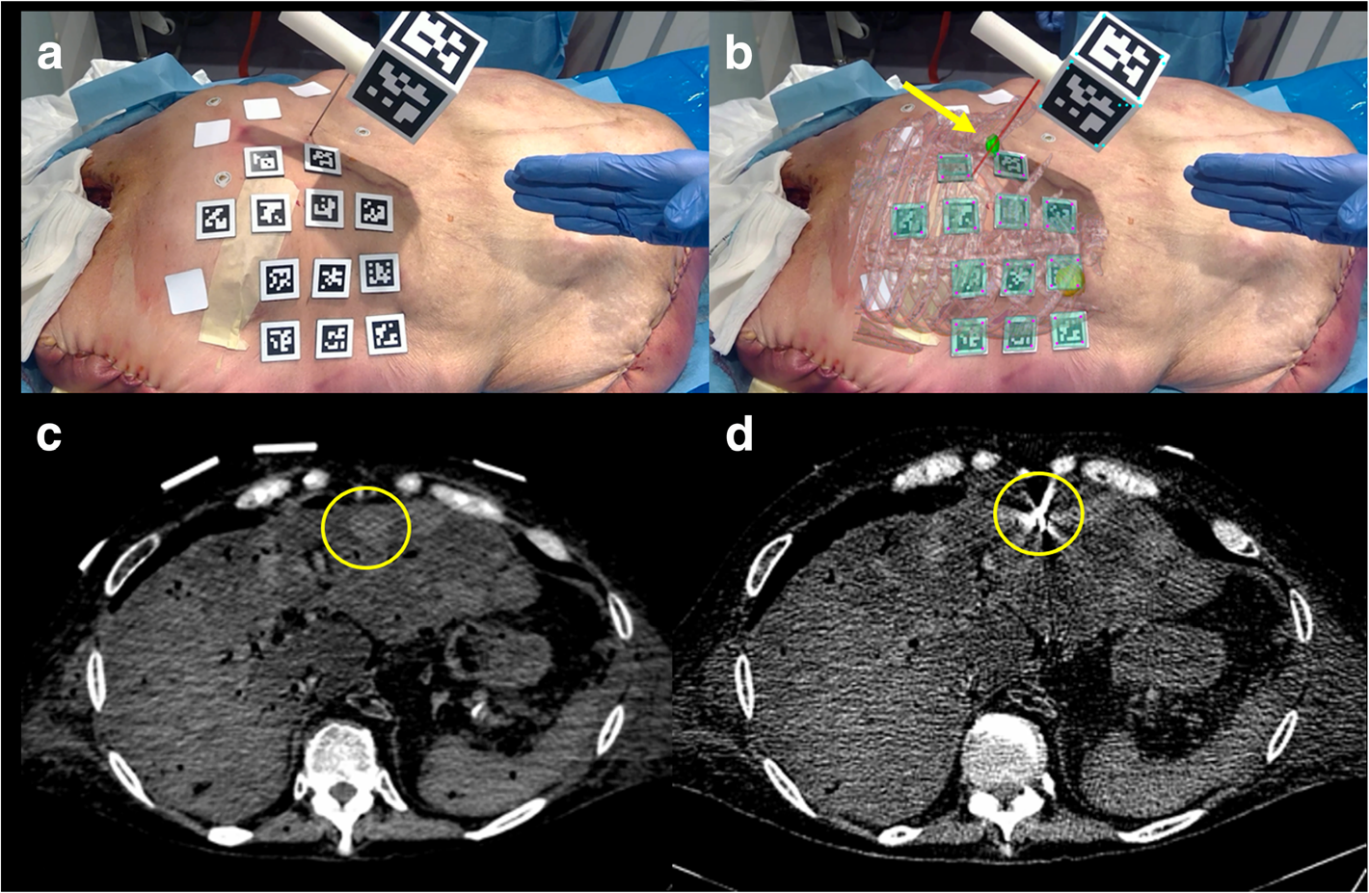
Cadaver model during the needle insertion in a liver metastasis (*yellow circle*) without (**a**) and with (**b**) augmented reality. The augmented reality shows the target in *green* and the needle in *red*. Correspondent axial CT images before (**c**) and after (**d**) needle insertion. The *yellow arrow* shows the target and the needle position in (**b**). The four *white squares* on the cadaver are medicated plasters and are not used for the augmented reality

**Fig. 7:**
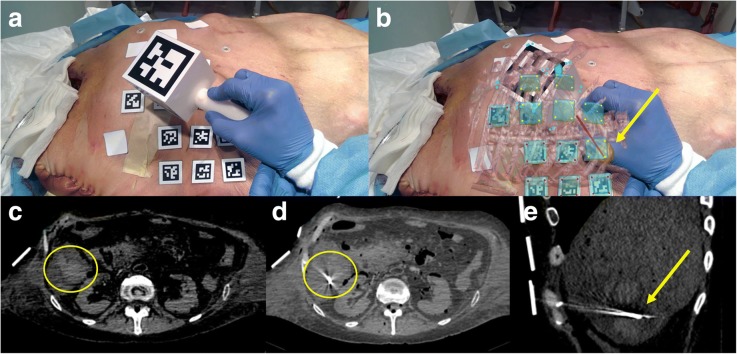
Cadaver model during needle insertion in a liver metastasis (*yellow circle*) without (**a**) and with (**b**) augmented reality. The augmented reality shows the target in *yellow* and the needle in *red*. Corresponding axial CT images before (**c**) needle insertion and axial (**d**) and sagittal (**e**) CT images after needle insertion. The *yellow arrow* shows the target and the needle position in (**b**). The four *white squares* on the cadaver are medicated plasters and are not used for the augmented reality

Based upon the US study, two liver metastases were selected as targets, one in segment IV (1.8 cm in size) and one in segment VI (3 cm). The cadaver was placed on its back on the CT gantry and a ventilator (Servo 900 C, Siemens, Erlangen, Germany) was attached to its trachea to induce simulated respirations. Twelve fiduciary surface markers were placed on the cadaver’s skin (Figs. 6a and 7a).

With the ventilator stopped midway in the respiratory cycle between inspiration and expiration, a CT scan of the cadaver was performed using a 16-slice unit (Emotion, Siemens Healthcare, Erlangen, Germany) with the following technical parameters: collimation = 3–5 mm; reconstruction interval = 2 mm; matrix = 512 × 512; in-plane pixel size = 0.48–0.86 mm; 130 kVp; 220 mAs; gantry rotation time = 0.5 s; and pitch = 0.784).

The volume of the targets (skin, bone, targets, markers) were then reconstructed using reconstruction proprietary algorithms. Subsequently, the needle was inserted in the cadaver using augmented reality guidance, until reaching the targets. The needle insertion was performed with the ventilator stopped in mid respiration to match the same respiratory phase of the initial CT scan until the target was reached on the augmented reality screen. Finally, CT scan of the cadaver with the inserted needle was performed. Accuracy of the final needle position was measured on CT images as for the pig model.

### Statistical analysis

Data are presented as mean ± standard deviation (SD) or as mean and range.

## Results

### Anthropomorphic chest model

The accuracy of the Endosight system measured ten times on the anthropomorphic model was, on average, 2.0 ± 1.5 mm (mean ± SD) without differences based upon the target rod selected, well below the 5 mm accuracy threshold set (Table 1). Overlapping of augmented reality on the bars coming out of the model is shown in Fig. 3.

**Table 1:** Results of anthropomorphic chest model accuracy test

	Real distance (average of 10 trials)	Virtual distance^*^ (average of 10 trials)	Absolute difference (real minus virtual distance)
Bar1-Bar2	13.5	12.0	1.5
Bar1-Bar3	28.0	29.3	1.3
Bar1-Bar4	76.0	78.5	2.5
Bar1-Bar5	184.5	181.5	3.0
Bar2-Bar3	15.5	17.0	1.5
Bar2-Bar4	64.5	66.3	1.8
Bar2-Bar5	167.5	170.5	3.0
Bar3-Bar4	48.4	47.2	1.2
Bar3-Bar5	156.0	157.5	1.5
Bar4-Bar5	104.5	106.8	2.3

All data are mm. The columns represent the distances in mm between each bar. The virtual distance between each bar was measured at three different tablet camera-phantom distances (300 mm, 400 mm and 500 mm) with the same results

^*^ Measured on augmented reality

### Porcine model

Without breathing control, the mean distance from geometric centre of the target to the needle tip was 8.0 ± 0.5 mm (mean ± SD), thus beyond the 5 mm accuracy threshold. However, with suspension of breathing, the mean distance from geometric centre of the target and the needle tip was 3.9 mm ± 0.4 mm (mean ± SD) (Figs. 4 and 5). As measured by CT, the depth of the kidney target with respect to the needle entry point was 107.6 mm, of the first liver target was 123.7 mm and of the second liver target was 92.5 mm.

The time required to set up the system was in the range of 4.5–6 min (mean = 5.3 min) and the time to perform each insertion was in the range of 6–8.5 min (mean = 7.2 min).

### Cadaver model

In the final experiment set up, performed with the next-generation, smaller-sized and differently shaped handle, accuracy, measured as the distance between the needle tip and the geometric centre of the target, was 2.5 mm for the first metastasis (Fig. 6) and 2.8 mm for the second metastasis (Fig. 7). Thus, both metastases were correctly targeted with the needle in the centre of the metastasis. The depth of the first liver metastasis with respect to the needle entry point was 50.6 mm and of the second liver metastasis was 91.2 mm.

The time required to set up the system was in the range of 5.0–6.2 min (mean = 5.8 min) and the time to perform each insertion was in the range of 8.7–10.8 min (mean = 9.4 min).

## Discussion

In clinical practice, image-guided percutaneous procedures, and in particular image-guided thermal ablations, are having a larger and larger diffusion, and are nowadays applied in a large variety of diseases in different organs, including liver, kidney, lung, and even thyroid and breast diseases [9–13]. The final result of all these treatments highly depends on the precision of the guiding system used during the procedure. This is the main reason why several efforts have recently been made not only to improve the efficacy of ablative devices, but also to increase the accuracy of image-guiding systems. Performance of procedures under advanced imaging modality guidance such as positron emission tomography/CT, improvement of US visualisation with application of intravenous contrast material, or simultaneous real-time fusion of different imaging modalities have been reported and used in order to improve the treatment result [2–4, 14].

In this scenario, the application of augmented reality to interventional procedures might have a relevant impact in further improving the precision of guidance during ablation and holds the potential for a large diffusion in the near future. Indeed, our investigation demonstrates that an augmented reality system can provide accurate guidance for interventional oncology procedures. In fact, under three different experimental settings, an accuracy < 5 mm was fulfilled with the exception of porcine model without breathing control.

The first experiment, performed on an anthropomorphic chest model, demonstrated that in a rigid system an accuracy of 2.0 ± 1.5 mm can be achieved, likely at the limit of imaging resolution. The second experiment, on a porcine model, clearly demonstrated the need for some form of breathing control to permit accurate fusion. In fact, leaving the pig free to breathe, it was not possible to obtain accuracy < 5 mm. Only when breathing control was performed were the accuracy results satisfactory. These results are in keeping with a wide number of reports using other forms of imaging fusion where respiratory errors were attributed as a key cause for error [15, 16].

Considering these results, we performed the last accuracy test on a cadaver model with breathing control. Endosight was able to guide needles to the metastases’ centres with an accuracy of 2.5 mm for the first metastasis and 2.8 mm for the second metastasis.

In other studies, the use of augmented reality as a guidance system has been reported [17, 18]. However, to our knowledge, this is the first time that focal liver lesion targeting was performed using augmented reality only.

Our results are very encouraging regarding the possibility of using Endosight as part of the clinical routine. With respect to other navigation systems [2, 14, 19], Endosight can potentially guide interventional procedures without the need for further real-time intraprocedure imaging (such as US or CT), thereby avoiding possible exposure to ionising radiation and the need for an additional co-registered modality. In addition, augmented reality permits to visualise on a tablet the 3D model of organs and targets superimposed upon the real patient and not on a different screen.

We readily note that our prototypical system is currently undergoing additional ongoing refinements. One potential further improvement could be the use of 3D goggles that would match the operator’s precise field of vision to the imaging findings including the target deeply embedded within the patient. Additionally, based upon the results obtained in the porcine model and in the cadaver, and thanks to a progressive software improvement, our future work will be aimed at minimising size and number of skin markers in order to improve visibility and cover less of the patient’s body and to make the marker placement process less time-consuming. Another improvement for use in a clinical scenario will be developing a breathing control algorithm to match marker reciprocal position during the CT scan and during the procedure in the normal respiration of the patient. Further, we are addressing the ergonomics of the handle and we will endeavour to improve accuracy by correcting needle bending that occurs during needle insertion inside the body by using fibre optics running along the needle. The addition of such technology will potentially permit detecting deformations and accordingly allow for updating the virtual needle position in real-time when bending of the real needle occurs.

In conclusion, we report upon an image-fusion augmented reality system that can achieve < 5-mm accuracy under multiple experimental conditions. Further assessment of this technology under clinical scenarios is likely warranted.

## Abbreviations

3D: Three-dimensional
CT: Computed tomography
MR: Magnetic resonance
US: Ultrasound
